# An Overview of Apple Varieties and the Importance of Apple Consumption in the Prevention of Non-Communicable Diseases—A Narrative Review

**DOI:** 10.3390/nu16193307

**Published:** 2024-09-29

**Authors:** Karina Mierczak, Anna Garus-Pakowska

**Affiliations:** Department of Nutrition and Epidemiology, Medical University of Lodz, 90-752 Lodz, Poland; karina.mierczak@stud.umed.lodz.pl

**Keywords:** apple, nutrients, polyphenols, phytochemicals, natural antioxidants

## Abstract

Non-communicable diseases such as cardiovascular diseases, cancers, diabetes, and asthma are increasingly common due to factors like industrialization, urbanization, fast-paced life, stress, sedentary lifestyle, and unbalanced diet in the 21st century. These chronic conditions are a global epidemic, being among the top causes of death worldwide. Preventing these diseases through a nutritious diet is crucial, and scientific studies suggest that appropriate fruit intake, particularly apples, can lower the risk of various health issues. Apples, rich in bioactive compounds, vitamins, minerals, and dietary fiber, offer numerous health benefits. Regular consumption of apples helps reduce the risk of atherosclerosis, coronary artery disease, heart attacks, and diabetes, and also provides anti-asthmatic and anti-allergic effects. Apples aid in detoxification, improve digestion, enhance skin, hair, and nail health, and offer protection against cancers, Alzheimer’s, and Parkinson’s disease. Apples have been a dietary staple for centuries, consumed in various forms like juices, sauces, and ciders. The reviewed article emphasizes the health benefits of apples, highlighting their role in preventing civilization diseases. It also discusses the characteristics of common apple varieties and the impact of thermal processing on their nutritional content.

## 1. Introduction

**Figure 1:**
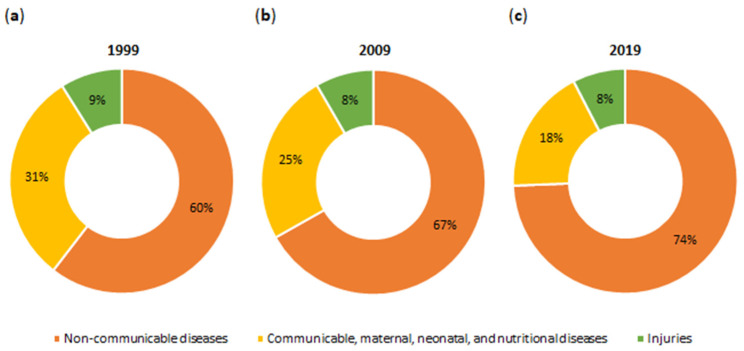
Global deaths by cause in (**a**) 1999, (**b**) 2009, and (**c**) 2019. The data pertain to both sexes and all age groups. Communicable diseases include infectious illnesses, such as malaria, tuberculosis, acquired immunodeficiency syndrome (AIDS). Injuries encompass accidents, homicides, suicides, and natural disasters [4,7].

The realities of the 21st century, such as industrialization, urbanization, population growth, fast pace of life, work overload, stress, sedentary lifestyle, and unbalanced diet, inevitably contribute to the development of numerous non-communicable diseases (NCDs). These include cardiovascular diseases, cancers, diabetes, and asthma [1,2,3]. Figure 1 shows NCDs among the global causes of death in 1999–2019. Since 1999, the number of deaths from NCDs has steadily increased (1999, 60%; 2009, 67%; and 2019, 74%) [4]. In 2019, among the top 10 global causes of death were ischemic heart disease, accounting for 16.17% of all deaths worldwide; diabetes mellitus, accounting for 2.74%; and colorectal cancer (CRC), accounting for 1.92% [5,6]. NCDs, commonly referred to as civilization diseases, are chronic conditions that occur all over the world. This explains why diseases in our civilization are called the epidemic of the 21st century [2,3].

A fundamental aspect of supporting human health is disease prevention through a nutritious diet. Food should provide an adequate amount of proteins, vitamins, and minerals. Scientific studies suggest that appropriate dietary fruit intake is associated with a reduced risk of a various adverse changes in the human organism [1,8].

**Figure 2:**
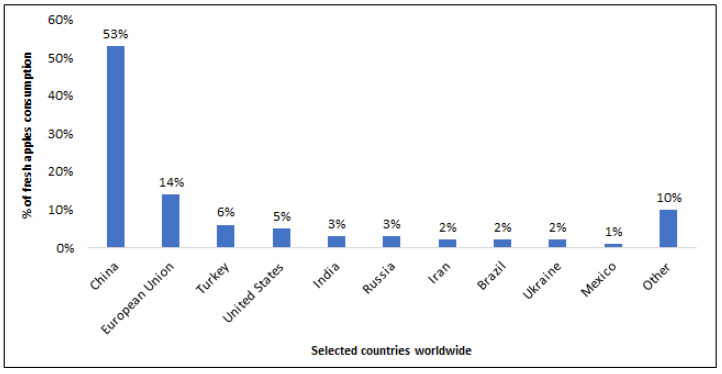
Global leading fresh apples consumption countries in 2022/2023 [9].

Among the numerous tree fruits grown in the world, one of the most popular is the apple. Apples and apple-derived foods have been consumed globally for many years due to their beneficial effects on human health. Apples hold varying degrees of popularity across different countries worldwide (Figure 2). In 2022/2023, the highest apple consumption, accounting for 53% of global consumption, was recorded in China. Comparatively, the European Union and Turkey consumed significantly fewer apples: 14% and 6%, respectively. This consumption trend has seen substantial growth over the past decade [9].

As the popular proverb says, “An apple a day keeps the doctor away”. Apples owe their reputation to numerous bioactive compounds, including phytochemicals, vitamins, minerals, and dietary fiber, the majority of which are present in both the flesh and the skin. These nutrients endow apples with distinctive properties [10,11]. Additionally, the aforementioned saying has received scientific confirmation. Gallus et al. studied individuals who consumed two apples per day and observed a reduced risk of cancer morbidity [12]. Moreover, an experimental study conducted on rats revealed that apple extracts, when mixed with drinking water and administered for 6 weeks, could diminish neoplastic lesions in rodents. The extract contained approximately 6 mg/kg body weight of procyanidins (PCs). These results led the authors to suppose that this dependence might also apply to the human organism. Consumption of two apples per day could provide a person the necessary amount of PCs, roughly 4–10 mg/kg body weight [13].

Consuming apples has a beneficial effect on the human organism in many aspects. The compounds present in these fruits help diminish the risk of atherosclerosis, coronary artery disease, and heart attacks [14]. Regular apple consumption also reduces the risk of developing diabetes and apples exhibit anti-asthmatic as well as anti-allergic properties [15]. Meta-analyses conducted by Gayer et al. for cardiovascular diseases, Guo et al. for type 2 diabetes mellitus, and Hosseini’s team for asthma provide evidence supporting these claims [15,16,17]. Apples aid in purifying the organism from toxins and remnants of undigested food in the intestines, thereby preventing diarrhea, bloating, and constipation [18]. Moreover, chewing apples helps eliminate bacteria in saliva [19]. Consumption of apples contributes to the de-acidification of organisms, enhances skin, hair, and nail conditions, and offers protection against various cancers, as well as Alzheimer’s and Parkinson’s disease [20,21,22,23,24]. Regarding cancer, a meta-analysis conducted by Fabiani et al. examined 41 studies and found that apple consumption significantly reduces the risk of CRC (OR = 0.56; *p* < 0.001) and breast cancer (OR = 0.79; *p* < 0.001) in case-controls for both types of cancers [25]. Research indicates that apple juice concentrate can enhance memory by regulating a crucial neurotransmitter, acetylcholine [26].

*Malus domestica* Borkhausen (Borkh.), *Rosaceae* family, genus *Malus*, stands as the most commonly cultivated apple tree in home gardens and plots. The official classification of one of the domesticated apple types is presented in Table 1. The *Rosaceae* family is divided into three subfamilies: *Amygdaloideae*, *Dryadoideae*, and *Rosoideae*. It is presumed that the primary ancestor of the apple is *Malus sieversii*, originating from Tian Shan forest in Central Asia [27].

*Malus domestica* Borkh. is a deciduous tree with broad branching. It can be either small- (2–6 m) or medium-sized (10–14 m). Short shoots, known as spurs, bear flowers and, subsequently, fruits. Fruits only develop on 1-year old shoots. The ovoid purplish buds are divided into vegetative buds, which form shoots or leaves, and flower buds, noticeably larger, blooming in April-May. The dark-green oval leaves have serrated edges, measuring 4–13 cm in length and 3–7 cm in width, arranged alternately. Flowers, with a diameter of 3 to 4 cm, consists of five sepals, five petals, and approximately 20 stamens, often growing in groups of 4–6. The spherical fruits come in various colors, such as red, yellow, or green, reaching a diameter of up to 15 cm. Their ripening period spans from August to October [27,31].

**Figure 3:**
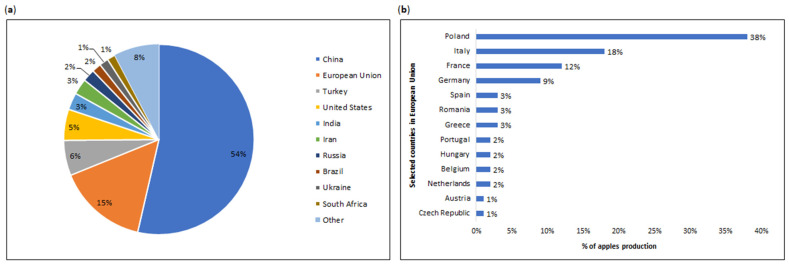
(**a**) World production of fresh apples in selected countries in 2022/2023 [9]. (**b**) Apple production in selected countries in European Union in 2022 (1000 metric tons (MT)) [34].

Apple trees are cold-resistant and bloom late. Optimal conditions for their development lie within the cool-temperature zone (35–50° latitude). These trees require sunlight, warm days, and cool nights. The growing season thrives under temperatures ranging 21–24 °C, with an annual rainfall of 100–125 cm. Loamy soils with pH of 5.5–6.5 are suitable for apple cultivation [27,32]. While apples typically thrive in temperate climates, they can also grow in varied environmental conditions, including tropical regions. In such cases, farming practices like irrigation become essential [27,33]. Consequently, major global producers include China, the European Union, Turkey, and the United States of America (USA), accounting for 54%, 15%, 6%, and 5%, respectively (Figure 3a) [9]. Within the European Union (Figure 3b), the dominant countries in the apple production are Poland, Italy, France, and Germany [34].

### 1.1. Apple Cultivars

There are approximately 7000 cultivars of *Malus domestica* known worldwide, such as *Royal Gala*, *Bravo*, *Granny Smith*, *Green Star*, *Fuji*, and *Golden Delicious* [33,35,36,37]. Many of these cultivars possess desirable traits suited for cultivation under specific conditions, varying in shape, size, color, aroma, and taste [27,33,35]. Malic acid and citric acid are the primary contributors to the acidity of apple fruit [38]. While most apple cultivars are diploid, some of them may be triploid (like *Jonagold*) or tetraploid (like *Antonovka Ploskaya*). However, all of them offer positive human health benefits [27,33,35]. Below is a brief description of selected species:

- *Royal Gala*, believed to have been discovered in 1934 in New Zealand, has *Kidd’s Orange Red* and *Golden Delicious* as its parent apples. Its non-uniform coloration showcases orange vertical strips with reddish blushes on a yellow skin, enclosing a dense white flesh.
- *Bravo*, bred in Western Australia from a cross between *Cripps Red* and *Royal Gala*, boasts a distinctive burgundy skin, making it a rich source of flavonoids.
- *Granny Smith*, an Australian cultivar named after “Granny” Anne Smith in 1868, emerges from a hybrid of *French Crab* apple and other cultivars, presenting a bright-green flesh.
- *Green Star*, originating from Europe, displays a bright-green, shiny skin enveloping a white flesh. It is a significant source of vitamin C and maintains its color even after being cut.
- *Red Delicious*, originating in the 1870s in Iowa, USA, displays a tall conical shape with dark red skin and creamy white flesh. *Golden Delicious*, hailing from West Virginia, USA, in 1912, features a large size, yellowish skin, and creamy white flesh.
- *Cripps Red* (also known as *Sundowner*) and *Cripps Pink* (*Pink Lady*) were bred by John Cripps in Western Australia in 1973 by crossing *Golden Delicious* and *Lady Williams*. *Cripps Red*, round-shaped with dark red skin and white lenticles, is both attractive and sweet, with ripeness enhancing its sweetness. *Cripps Pink* displays pink blush on a greenish-yellow skin base.
- *Braeburn*, discovered in New Zealand in 1952 from a cross between *Lady Hamilton* and *Granny Smith*, exhibits red-to-orange vertical strips on a greenish base.
- *Fuji*, a renowned Japanese cultivar, typically presents a round, large size, with a red-pink blush on a greenish-yellow base.
- *Lady Williams*, obtained in the 1930s in Donnybrook, Western Australia, originated from a cross between *Granny Smith* and *Jonathan.*

**Figure 4:**
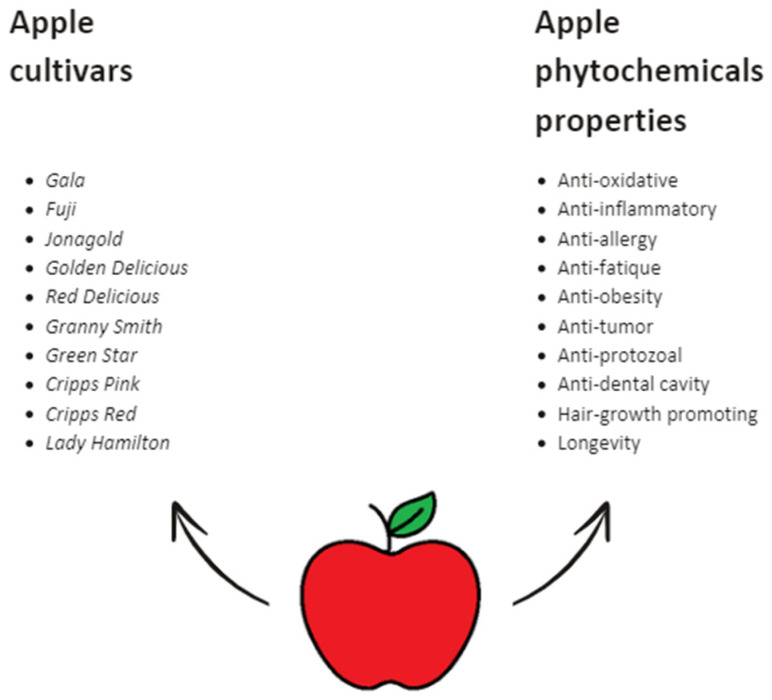
The diversity of apple cultivars and the health-promoting properties of apple phytochemicals [10,11,33,35,36,37].

Generally, most apples feature firm flesh, contributing to their sweetness, flavor, and juiciness. *Granny Smith* offers a sharp-tart taste, *Braeburn* a sweet-tart flavor, and *Fuji* a honey-sweet profile. Despite its green skin, *Green Star* is cultivated for its sweet taste [35]. In any case, regardless of the apple tree variety, all cultivars possess valuable properties beneficial for human health (Figure 4) [10,11,33,35,36,37].

### 1.2. Diseases of Apples

Various fungi and certain bacteria may cause apple infections, leading to diseases like scab, blue mold, brown rot, and Bull’s eye rot [39,40,41,42,43]. Apple scab is caused by the fungus *Venturia inaequalis*, while blue mold indicates the presence of *Penicillium expansum* [39,40]. Fungi in the *Monilinia* genus cause brown rot, and *Neofabraea* species cause Bull’s eye rot [41,42]. Additionally, fire blight, caused by the bacterium *Erwinia amylovora*, can affect fruits, leaves, or even the entire tree, resulting in significant yield losses. Hence, prioritizing the freshness of apples becomes crucial, opting for ripe and fragrant fruits [39,40,41,42,43].

Apples have been a dietary staple for centuries. Over time, more and more ways to consume them have appeared. Today, apples can be enjoyed in various forms, such as juices (with the greatest variety), sauces, or ciders [44]. The review article focused on the beneficial influence of chemical compounds found in apples. The aim of this review was to emphasize the value of consuming apples due to their potential benefits in preventing common diseases such as cancer or obesity.

## 2. Bioactive Compounds

Phytonutrients represent natural plant compounds with biological activity, some characterized by vibrant colors and distinctive aromas. Within plants, these compounds constitute a natural barrier against various external factors, including protection from ultraviolet (UV) radiation. In humans, phytonutrients strengthen the immune system and many of them have antioxidant properties, such as vitamin C and flavonoids. Phytochemicals protect the human organism against the harmful effects of free radicals and cellular degeneration, thereby playing a crucial role in preventing numerous chronic diseases, like cardiovascular diseases, diabetes, and even cancers. Moreover, they might help in reducing premature aging. Apples are a rich source of many essential phytonutrients, such as vitamin C, potassium, calcium, and dietary fiber. They boast a low sodium content (<1 mg), proving beneficial for patients managing conditions like arterial hypertension, chronic kidney disease, and cirrhosis. Incorporating apples into a low-sodium diet could potentially protect against heart attacks or strokes [35,45,46,47,48,49].

The content of phytochemicals in apples may vary based on the variety, ripening stage, season, and storage conditions of the fruit. For instance, cold storage conditions might lead to a reduction of up to 50% in total phenol content in the flesh and 20% in the peels. This occurs because phenolics are inherently unstable and undergo metabolic changes during cold storage [10,11].

### Apples as a Source of Antioxidants and Their Health Significance

**Figure 5:**
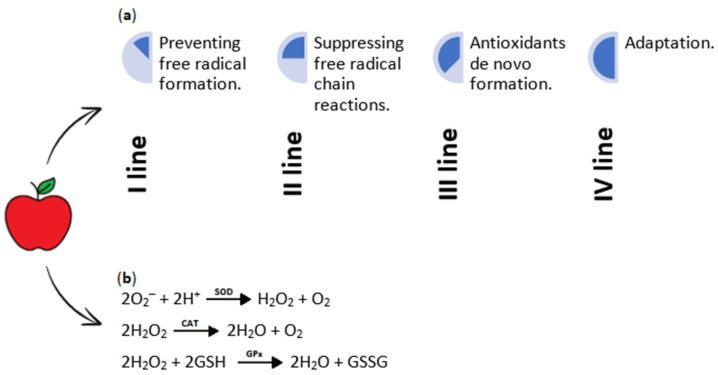
(**a**) Principles of antioxidant activity. Antioxidants function through four distinct mechanisms, commonly called the “lines of defense”. The first line of defense is preventive, where antioxidants inhibit the formation of free radicals. The second line of defense involves radical scavenging, where free-radical chain reactions are suppressed. The third line of defense concerns repair and *de novo* formation of antioxidants, involved in removing oxidatively modified molecules. Lastly, the fourth line of defense, known as adaptation, triggers the generation and response of free radicals, facilitating the production and delivery of required antioxidants to targeted sites. (**b**) Reactions of selected free radicals are catalyzed by superoxide dismutase (SOD), catalase (CAT), and glutathione peroxidase (GPx) enzymes. The primary enzyme responsible for neutralizing free radicals is SOD (EC 1.15.1.1), which catalyzes the breakdown of superoxide anions into hydrogen peroxide and oxygen. CAT (EC 1.11.1.6) reduces hydrogen peroxide to water and oxygen, while GPx (EC 1.11.1.9) reduces hydrogen peroxide to water [22,53,54,55]. O_2_^−^—superoxide anion; H^+^—hydrogen ion; SOD—superoxide dismutase; H_2_O_2_—hydrogen peroxide; O_2_—oxygen molecule; CAT—catalase; H_2_O—water molecule; GSH—reduced form of glutathione; GPx—glutathione peroxidase; GSSG—oxidized form of glutathione.

Numerous compounds found in apples act as antioxidants (Figure 5a), playing a crucial role in neutralizing free-radicals and reactive oxygen species (ROS). The natural presence of free radicals within cells is associated with aerobic metabolism. Antioxidants exert a protective effect on cells by impeding the formation of oxidative stress, which proves significant in various inflammatory diseases [22,50,51]. Interestingly, among fruits consumed in the USA, apples rank second in antioxidant capacity, following cranberries [50]. The study from 2021 has demonstrated that the oxygen radical antioxidant capacity (ORAC) value can reach up to 10,682.4 ± 1965.4 µM Trolox equivalents (TE) for pure apple [52]. Free radicals, such as hydrogen peroxide (H_2_O_2_), superoxide anion (O_2_^−^) (Figure 5b), and hydroxyl radical (^•^OH), are produced due to oxidation processes and have the potential to damage cells, deoxyribonucleic acid (DNA), and proteins, consequently contributing to aging and various diseases such as cardiovascular diseases, neurodegenerative disorders, autoimmune conditions, and cancers. Furthermore, antioxidants might minimize the oxidation of low-density lipoprotein (LDL). This prevents the accumulation of LDL on cell membrane surfaces within blood vessels, thereby averting the narrowing of vessel lumens. As a result, antioxidants aid in preventing atherosclerosis, circulatory issues, and, ultimately, heart failure [35,51].

The most prevalent non-enzymatic antioxidants include vitamin C (ascorbic acid), which is a monosaccharide and functions as a hydrophilic antioxidant [22]. Vitamin C exists in cells primarily in its reduced form (i.e., ascorbate), which plays a role in reducing and neutralizing ROS. The presence of vitamin C protects against UV damage and stimulates collagen synthesis, contributing to the maintenance of healthy skin, hair, and nails [21,35,51]. Ascorbate is vital for maintaining balance and proper functioning within the central nervous system. It acts as a key neuroprotective agent by hindering the attachment of the glutamate neurotransmitter to synaptic receptors. Additionally, it modulates the function of glutamate receptors and reduces the activation of *N*-methyl-D-aspartate (NMDA) receptors. These actions effectively shield neurons from the detrimental effects of glutamate excitotoxicity [51].

**Phytonutrients** **→ Polyphenols**

The overall apple polyphenol content ranges from 0.01% to 1% in the fresh fruit [56]. Polyphenols (e.g., catechin, chlorogenic acid) may affect epigenetic mechanisms, including DNA methylation, histones modification, and micro ribonucleic acid (miRNA) expression. Moreover, they promote the activity of other antioxidants, especially glutathione, ascorbic acid, α-tocopherol, and the previously mentioned enzymes involved in free-radicals neutralization. Phenols occur in many plant organs, including roots, stems, leaves, flowers, and fruits. They are prominently present in both the flesh and peel of fruits, with the peel containing the highest abundance of polyphenols. This distribution aids in shielding the plant against harmful UV radiation [10]. Exposure to light beams can trigger partial oxidation of phenols, resulting in color changes, such as pink or brown. The same reaction occurs during apple ripening [57,58]. The natural enzymatic browning of apples is a result of different levels and forms of polyphenolic compounds [57,58,59]. Polyphenol oxidase (PPO), an enzyme located in chloroplasts, plays a pivotal role in this process [60]. Initially, in the presence of oxygen, monophenols are hydroxylated to o-diphenols. This reaction is catalyzed by monophenolase or cresolase. Subsequently, diphenolase or catecholase oxidizes o-diphenols into o-dichinones. These o-dichinones, highly reactive compounds, polymerize and generate yellow, pink, red, or brown pigments [57,58,59]. Ascorbic acid acts as a natural inhibitor of PPO [61].

**Figure 6:**
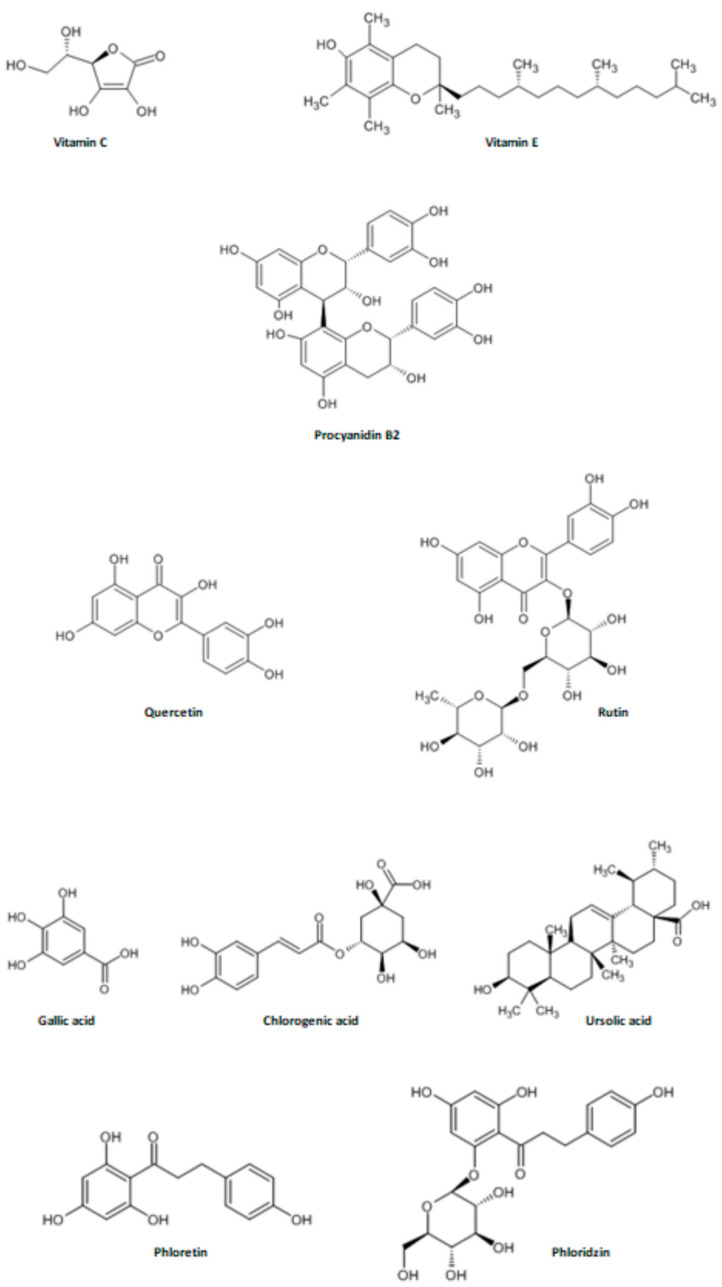
The structural formulas of selected apple antioxidants [64].

Furthermore, the type of polyphenol determines the color, taste, and metabolic activity of the plant it originates from [62]. Their content also varies among apple cultivars. Flavonoids (e.g., procyanidins, catechins, epicatechins, quercetin, rutin, gallic acid, chlorogenic acid, phloridzin (Phldz) (Figure 6) are the most abundant group of polyphenols present in apple flesh. Interestingly, even higher concentrations are observed in apple peels and in darker, redder apples. These flavonoids act as chelators for metal ions [35,63].

Understanding the biological activity and metabolism of phenolic compounds is crucial for comprehending their potential pro-health properties. Approximately 67–100% of apple phenolic compounds are metabolized in the small intestine, while around 30% in the colon among ileostomy patients [62,65]. However, in healthy individuals, different proportions are observed: 48% of polyphenols are digested in the small intestine and 42% in the colon, while 10% remain undigested [66]. Furthermore, phenolic compounds undergo diverse metabolization processes, impacting their bioavailability for human cells. Studies reveal that 12.7% of phenols from apple juice remain unmetabolized in the intestine, with 22.3% existing in the form of metabolites [62,65]. Polyphenols also enhance the activity of mitochondrial dehydrogenases. Research by Masuda et al. demonstrated that polyphenols’ presence promoted the expression of the *proliferator-activated receptor gamma coactivator 1-alpha* (*PGC-1*α) gene, consequently boosting mitochondrial DNA levels in murine chondrocytes. Moreover, they improved mitochondrial depolarization, compensating for the lack of superoxide dismutase 2 (SOD2), an essential component of each mitochondrion and vital in mitigating oxidative stress. Additionally, polyphenols have been linked to the regulation of myosin heavy chain isoforms, contributing to increased muscle strength and reducing the incidence of arrhythmias [67]. Practically, various methods are available to measure polyphenolic compound levels in apple extracts. Techniques such as high-performance liquid chromatography, hydrochloric acid (HCl)-butanol assay, and the Folin–Ciocalteu assay are commonly used for polyphenol quantification [68,69].

**Phytonutrients** **→ Polyphenols** **→ Flavonoids** **→ Procyanidins**

Procyanidins present in apples, mostly B1, B2, and C1, are a mixture of various monomers of (+)-catechins and (−)-epicatechins interconnected by characteristic bonds [67]. The activity of PCs depends on the degree of their polymerization [70]. In a comparative analysis by Hammerstone et al., varying amounts of PCs were observed in different apple varieties. *Red Delicious* exhibited the highest content, with 207.7 mg/portion, while *Golden Delicious* showed the lowest, at 92.5 mg/portion. Interestingly, higher procyanidins concentrations were detected in the skin compared to the pulp, and their content decreased with fruit maturity [71].

These compounds play a significant role in the circulatory and skeletal systems, impacting related diseases, such as osteoarthritis. PCs, notably, have a positive effect on the cartilage homeostasis. Procyanidin B2 regulates aggrecan synthesis, which is the most important proteoglycan predominant in the extracellular matrix of chondrocytes in cartilage. Aggrecan protects cartilage tissue against abrasion, harshness, and other forms of damage, maintaining its mechanical strength and viscoelasticity. Studies by Masuda et al. revealed a correlation between the presence of polyphenols, matrix-related genes, and increased aggrecan gene expression levels. This positive effect on proteoglycan synthesis was observed in the presence of procyanidins, concurrently decreasing the expression levels of Matrix Metallopeptidase 3 and 13 [67].

Scientific finding has shown the inhibitory impact of PCs on pancreatic lipase activity, thereby reducing triglyceride absorption in the small intestine. Under normal conditions, pancreatic lipase hydrolyzes triglycerides into glycerol and fatty acids, which are subsequently absorbed into the bloodstream. This process indirectly contributes to triglycerides’ accumulation in the liver and the onset of obesity. A study has demonstrated that the greater the degree of polymerization of procyanidins, the stronger the inhibitory effect of pancreatic enzymes [72].

Toda et al. indicated that PCs exhibit inhibitory effects on the progression of neurodegenerative diseases, including Alzheimer’s disease (AD) [23]. AD is characterized by extracellular amyloid-β (Aβ) aggregates (i.e., amyloid plaques, neuritic plaques) and the presence of tau protein inside neuronal cells. The Aβ peptide, derived from the amyloid precursor protein, stems primarily from protein misfolding, leading to irreversible changes that cause neuronal cell death, impairing memory and other intellectual skills [73]. According to Toda et al., PCs, depending on the concentration, prevent β-amyloid aggregation, particularly amyloid-β 42 (Aβ42), commonly localized in the core of senile plaques, showcasing potential neuroprotective properties [23]. Additionally, phytochemicals have been observed to downregulate presenilin-1 expression (intramembranous protease), a catalytic subunit of γ-secretase responsible for the Aβ generation [10,74].

Baldness, a common cause of hair loss, is an issue impacting many individuals. Reports indicate that proper nutritional intake plays a significant role in maintaining healthy hair, skin, and nails. Specifically, 1% procyanidin B2 might support hair growth and volume. Procyanidin B2 has an additional effect in conjunction with some vitamins, especially A, B, and C, that are vital for healthy scalp and preventing dandruff [75].

**Phytonutrients** **→ Polyphenols** **→ Flavonoids** **→ Quercetin**

Quercetin (3,3′,4′,5,7-pentahydroxyflavone) is a glycoside present in the apple peel [76,77]. Its level in apples typically reaches 2.1–7.2 mg/100 g. The dominant forms of quercetin in apples are quercetin O-arabinoside, 3-O-galactoside, 3-O-glucoside, and 3-O-rhamnoside. The antioxidant properties of quercetin stem from its ability to act as a hydrogen donor [77]. Quercetin deficiency may potentially be related to cardiovascular diseases, like hypertension. Studies suggest that quercetin has the capability to enhance nitric oxide (NO) activity through elevating the expression of endothelial NO synthase and suppressing the activity of enzymes responsible for NO degradation (e.g., nicotinamide adenine dinucleotide (phosphate) hydrogen (NAD(P)H) oxidase and arginase). NO, in turn, widens blood vessels, leading to increased blood flow and decreased blood pressure [35,78].

Quercetin not only induces the relaxation of smooth muscle within the cardiovascular system but also prevents platelet aggregation [77]. Additionally, various studies have shown the inhibitory effect of quercetin on sulfotransferases, enzymes engaged in the modulation of thyroid hormones [10,79]. Recent evidence indicates that quercetin may play a role in combating viral infections, including but not limited to influenza-A virus, hepatitis B and C viruses, human immunodeficiency virus (HIV), dengue virus, and Japanese encephalitis virus [78].

**Phytonutrients** **→ Polyphenols** **→ Flavonoids** **→ Rutin**

Apples contain flavonol 3,30,40,5,7-pentahydroxyflavone-3-rhamnoglucoside, commonly known as rutin, rutoside, rutinum, sophorin, or vitamin P, which is a glycoside of quercetin [63,80]. Total rutin content present in apples may vary between 3.69 ± 1.34 and 374.50 ± 2.35 mg/100 g of dry extract weight, depending on the cultivar [81].

Rutin has potential antidepressant effects by increasing serotonin and noradrenaline levels in the synaptic cleft. Moreover, rutin demonstrates protective qualities against cataracts. It reduces intramolecular pressure and levels of malondialdehyde (MDA), a byproduct of lipid peroxidation that indicates the presence of ROS in the organism. Elevated MDA levels contribute to disruptions in cell membrane integrity. Rutin has shown protective effects against damage induced by lipid peroxidation in human sperm. Furthermore, it exhibits antibacterial properties against organisms like *Escherichia coli*, *Proteus vulgaris*, and *Pseudomonas auruginossa*, and possess antiviral and antifungal capabilities against *Candida gattii* [63].

**Phytonutrients** **→ Polyphenols** **→ Flavonoids** **→ Other**

Among other noteworthy polyphenols, phloretin (Phlor), phloretin-20-O-xyloglucoside, and its glycoside Phldz, phloretin 20-O-glucose, are gaining special attention. These compounds are found in apple flesh (0.4–0.8 µg/g fresh weight (FW) of Phlor; 6.6–45.1 µg/g FW of Phldz) and peel (0.6–2.2 µg/g FW of Phlor; 16.4–84.11 µg/g FW of Phldz). Phlor, a hydrolyzed form of Phldz, belongs to the dihydrochalcones class of phenylpropanoids. It consists of two aromatic rings connected by a C3 chain, along with a β-D-glucopyranose moiety. Study indicates that Phlor reduces the synthesis of proinflammatory molecules, like prostaglandin E2 and interleukin 8, which regulate the chemical attraction, infiltration, and adhesion of immune cells at inflammatory sites. Additionally, Phlor has demonstrated dose-dependent inhibition (ranging from 0.01–1.0 mM) of advanced glycation end products, associated with age-related disorders. Phlor also augments the antioxidative activity of glutathione in Caco-2 and HT-29 colon cancer cells. Studies have reported the beneficial effects of Phlor and Phldz in mitigating inflammation in the lungs, liver, and intestines [82].

**Phytonutrients** **→ Polyphenols** **→ Triterpenes**

Triterpenes, a wide group of natural compounds synthesized from basic isoprene units, can be converted into other metabolites, like sterols, steroids, and saponins. Terpenes, hydrocarbons, give rise to triterpenoids upon oxygenation (oxygen derivatives of terpenes), though both terms are often used interchangeably despite slight differences in their properties. Triterpenes are divided into six classes: monocyclic, bicyclic, tricyclic, tetracyclic, pentacyclic, and acyclic [83]. Apple phytochemicals encompass a broad spectrum of triterpenoids, all of which exhibit potential anticancer properties against various types of cancer cell lines. Notable triterpenoids found in apples include 2α-hydroxyursolic acid, 2α,3β,13β-trihydroxyurs-11-en-28-oic acid, 3β,13β-dihydroxyurs-11-en-28-oic acid, 3β,28-dihydroxy-12-ursene, 3β-hydroxyurs-12-en-28-oic, and 3β-trans-p-coumaroyloxy-2α-hydroxyolean-12-en-28-oic acid [33,84].

**Phytonutrients** **→ Polyphenols** **→ Triterpenes** **→ Ursolic acid**

3β-hydroxyurs-12-en-28-oic, recognized as ursolic acid (UA), is a lipophilic pentacyclic triterpene prominently found in the waxy peel of apples, such as *Red Delicious*, where concentrations can reach up to 248.02 ± 0.08 µg/mL [33,85,86].

Ursolic acid exists in two isomeric forms: oleanolic and betulinic acids [33,87]. It occurs as a free acid or as glycosides non-saccharide component (aglycone) of triterpene saponins. UA exhibits inhibitory potential against various Gram-positive bacteria species (e.g., *Staphylococcus aureus*, *Methicillin-resistant Staphylococcus aureus* (MRSA), *Mycobacterium tuberculosis*) and protozoans (e.g., *Plasmodium falciparum*, *Leishmania major*, *Trypanosoma brucei*). In *Plasmodium falciparum*, UA acts on the calcium ions (Ca^2+^) pathway, while in *Trypanosoma brucei*, it impedes glyceraldehyde 3 phosphate dehydrogenase (GAPDH) activity. This action helps prevent the formation of bacterial biofilm, offering protection against multiple infections. UA is highly regarded for its anti-inflammatory activity, owing to its ability to inhibit the transcription factor nuclear factor kappa B (NF-κB), regulating the expression of numerous anti-inflammatory response genes. Additionally, UA can impede selectin E expression, commonly found on the surface of endothelial cells during inflammation [33].

## 3. Apples and Their Anti-Cancer Potential

Cancer poses a significant global health challenge, with approximately 20 million reported instances in 2022, impacting 10,306,455 of men and 9,658,356 of women worldwide [88].

The treatment of carcinoma often follows a similar approach based on several factors, such as the disease stage, tumor location, presence of metastases, overall health of the patient, and patient preferences. In early-stage carcinoma treatment, this may involve surgical procedures to remove the cancerous tumor along with surrounding tissues or organs, and in some cases, organ transplants. Advanced cases may necessitate partial or complete removal of the affected organs. Cancer treatment methods also encompass radiation therapy, utilizing radiation to eradicate cancer cells. It can be used pre-surgery to reduce tumor size, post-surgery to eliminate any remaining cancer cells, or as a primary treatment for advanced cases. Chemotherapy, the administration of anti-cancer drugs, remains a central treatment aiming to destroy or impede the growth of cancer cells. Occasionally, chemotherapy is given before or after surgery [89].

Innovative approaches in cancer treatment, such as immunotherapy-based combinations, e.g., tremelimumab with durvalumab or nivolumab with ipilimumab for hepatocellular carcinoma (HCC), are now emerging as preferred options for initial therapy [90]. These alternative treatments aid in shrinking the tumor, slowing its progression, and alleviating associated symptoms [89].

Apples boast an abundance of compounds renowned for their cytoprotective and anti-cancer properties. These elements possess the capability to delay and safeguard cells against the onset of carcinogenesis [91]. The collective findings suggest that regularly consuming apples can help prevent against the onset and progression of various types of cancer, such as colorectal cancer and lung cancer [92,93,94].

Chemoprevention broadly refers to the use of natural or synthetic substances to impede the carcinogenesis process and the progression of benign tumors to malignant ones [89]. Some in vitro studies show promising indications of certain bioactive compounds found in apples in relation to their potential anti-tumor properties [93,95,96,97]. Understanding the relevant molecular targets allows for the exploration of apple-derived compounds in developing tailored drug formulations and therapies [89].

### 3.1. Human Hepatocellular Carcinoma

Hepatocellular carcinoma ranks among the most prevalent and fatal forms of malignancies worldwide [98]. In 2022, approximately 865,269 cases of liver cancer were diagnosed globally, resulting in 757,948 deaths [88].

Diagnoses of HCC frequently occur at advanced stages of disease, leading to an overall unfavorable prognosis. The cancer develops due to underlying dysfunction in the liver, with primary risk factors including chronic hepatitis B and hepatitis C infections. Other contributing factors comprise consumption of fungal toxin aflatoxin B1-contaminated foods, excessive alcohol intake, non-alcoholic steatohepatitis, cirrhosis, diabetes, obesity, and hereditary hemochromatosis [98].

Phloretamide (PA), a compound derived from apples, has shown potential in protecting against the development of liver cancer. As a derivative of phloretic acid, which results from phloretin metabolism, PA stimulates the nuclear factor erythroid 2-related factor 2 (Nrf2), along with its target genes responsible for antioxidative and detoxifying activities. This process involves the activation of enzymes, such as glutathione S-transferase, NAD(P)H quinone oxidoreductase 1, and heme oxygenase 1. Consequently, PA exhibits the potential to mitigate the carcinogenic effects induced by factors, like oxidative stress or excessive free radicals [95].

The Nrf2 protein is encoded by the *nuclear factor erythroid-derived 2-like 2* (*NFE2L2*) gene [99]. Typically, Nrf2 exists in an inactive form within the cytoplasm, serving to maintain cellular genome stability. However, under certain conditions, it can bind to specific DNA sequences. The Nrf2-antioxidant response element (ARE) pathway is notably significant, often targeted by chemo-preventive agents. In the case of PA, depending on its concentration, it prompts the translocation of Nrf2 from the cytosol to the nucleus. There, it binds to ARE. Interestingly, increased Nrf2 expression was observed exclusively in normal adult liver cells. Scientific studies have proved that PA has preventive effects against liver cancer. While low concentrations of PA did not impact Nrf2 expression in human liver cancer cells like HepG2, concentrations of 100–200 µM proved cytotoxic to HepG2 cells [95].

### 3.2. Colorectal Cancer

Colorectal cancer ranks as the third most prevalent cancer globally and the second most lethal, leading to approximately 1,926,118 new cases and 903,859 deaths worldwide in 2022. Its occurrence is higher in highly developed countries compared to low-income ones [88].

CRC encompasses both colon and rectum cancer [96]. Colon cancer often correlates with the aggressiveness of colon polyps, small protrusions originating from mutated epithelial cells [65]. The morbidity in CRC patients is influenced by genetic and environmental factors (e.g., ageing population and lack of physical exercises) [100,101].

Apple phytochemicals exhibit chemo-preventive properties against CRC. Polysaccharides from apples have been shown to minimize DNA damage and abnormal lesions like aberrant crypt foci in the colon or rectum, inducing apoptosis in cancer cells. These compounds also inhibit specific cellular pathways in human CRC cells, such as HT-29 and Caco-2 [65,96,102]. Apple polysaccharides (at doses of 0.001–0.1 mg/mL) can arrest the G0/G1 phase of the cell cycle in HT-29 cells [96]. Studies show that quercetin, found in apples, may inhibit signaling pathways in colon carcinoma cells [96,103,104]. This compound inhibits epidermal growth factor receptor (EGFR) autophosphorylation in HT-29 cells and deactivates extracellular signal-regulated kinases 1 and 2 (ERK1/2), and mitogen-activated protein kinases (MAPK) [96,103]. Quercetin also decreases Bcl-2 levels by inhibiting NF-κB [96,104]. Moreover, combining quercetin with another polyphenol, trans-pterostilbene (trans-3,5-dimethoxy-4′-hydroxystilbene), along with chemotherapy and radiotherapy, resulted in the destruction of 85% of HT-29 cells, leading to long-term survival (>120 days) [104]. Additionally, apple polyphenols are involved in cellular signaling pathways committed to the progression of various types of cancers like the phosphoinositide 3 kinase (PI3K)/protein kinase B (Akt) pathway [105,106].

Naturally occurring chemical compounds can enhance the effects of specific drugs. For example, oligogalactan (OG) may influence the toll-like receptor 4 (TLR-4)/NF-κB pathway. Li et al. demonstrated in in vitro studies that OG from apples strengthened the inhibitory action of 5-fluorouracil (5-FU) on 2 CRC cell lines: HT-29 and SW-620. OG intensifies 5-FU-induced cell apoptosis and inhibits the S phase by downregulating cyclin A and cyclin-dependent kinase 2 expression. Furthermore, OG demonstrates a protective role in cases of intestinal toxicities [107].

### 3.3. Breast Cancer

In 2022, there were 2,308,897 newly diagnosed cases of female breast cancer, resulting in approximately 665,684 fatalities [88].

Breast cancer is characterized by the uncontrolled growth of cells within the breast tissue. Regular breast self-exams, routine mammograms, and maintaining a healthy diet contribute to reducing the risk of developing the disease [108].

Triterpenoids found in apples may offer protective benefits against breast cancer [109]. Triterpenoids belong to isoprenoids derived from its precursor, squalene [97]. 3β-trans-cinnamoyloxy-2α-hydroxy-urs-12-en-28-oic acid (CHUA) is a representative triterpenoid encountered in apple peels’ cuticular waxes and has demonstrated anticancer properties [109]. In this form, triterpenoids serve multiple roles, including influencing fruit susceptibility to microbes and external factors, minimizing water loss, and preserving nutrients. The triterpenoid concentration in fruit peels often fluctuates with ripeness, presumed to offer photoprotection, especially in colorless fruit types, complementing the role of various polyphenols [97,109].

In vitro studies have proved that in CHUA-treated MDA-MB-231 cells, DNA fragmentation was clearly visible. Such fragmentation is characteristic of the apoptosis process, indicating the CHUA’s action via mitochondrial pathways. This evidence suggests that CHUA disturbs mitochondrial transmembrane potential and activates apoptosis in human breast cancer cells by promoting pro-apoptotic proteins (i.e., Bax, Bcl-2), thereby boosting mitochondrial membrane permeability. The balance between pro- and anti-apoptotic factors defines cell survival. Notably, the findings of Qiao et al. have proved that the best inhibitory effect of CHUA half-maximal inhibitory concentration (IC_50_)~2.57 ± 0.11 µM) was reached for the MDA-MB-231 cell line (an epithelial human breast cancer cell line) [109]. Dashbaldan et al. compared the content of different triterpenoid compounds in different fruits over 4 consecutive months. Their results highlighted *Malus domestica* as the richest in neutral triterpenoids (like amyrin, botulin, and uvaol) and triterpenoid acids (like oleanolic acid, betulinic acid, and ursolic acid) in September [97].

### 3.4. Lung Cancer

Lung cancer is one of the most serious and leading causes of death worldwide. In 2022, there were 2,480,301 reported new cases, representing 12.4% of all newly diagnosed cancer cases and ranking first of all cancers. Correspondingly, the mortality rate for lung cancer in the same year stood at 1,817,172 deaths, constituting 18.7% of all cancer-related fatalities [88].

Lung cancer arises within the lungs and can spread to lymph nodes or other organs throughout the organism. Several factors, including exposure to tobacco smoke, ionizing radiation, and viral infections, can contribute to its development [110].

Interestingly, there is evidence to suggest that consuming apples may potentially reduce the risk of lung cancer development or progression. Studies have indicated a decreased risk of lung cancer among women who ate one apple more than before [93,94]. On the contrary, other study recorded that eating apples is closely related with cancer risk in men more than in women. The difference might be linked to the higher prevalence of smoking among men than women, which can contribute to oxidative damage in the lungs [25]. Antioxidants present in fruits like apples might nullify these debilitating changes [94]. Additionally, quercetin found in apples might arrest cytochrome P450 enzymes in lung cancer, such as cytochrome P450 family 1 subfamily A member 1 (CYP1A1), which could be responsible for the induction of carcinogenesis [111].

### 3.5. Oral Squamous Cell Carcinoma

One of the most common types of oral cancer is oral squamous cell carcinoma (OSCC). Oral squamous cell carcinoma is a malignancy growth that can manifest in any part of the oral cavity. It exhibits local invasiveness, with occasional spread to nearby lymph nodes, and occasionally metastasizes to distant areas [112]. In 2022, there were 389,485 reported cases of OSCC, resulting in 188,230 fatalities [88].

OSCC expands in a similar way in humans and rats. Ribeiro et al. have analyzed the correlation between apple extracts and the gene expression of antioxidant enzymes (i.e., SOD2—mitochondrial enzyme, copper zinc superoxide dismutase (CuZnSOD)—cytoplasmic enzyme). 4-nitroquinoline 1-oxide, which has the ability to form DNA adducts, was used to induce OSCC in the rat tongue. The increased SOD2 and CuZnSOD expression levels in cancer cells after treatment with apple extract suggests that apple compounds minimize oral carcinogenesis [113].

## 4. Weight Management: Overcoming Obesity

Obesity is a critical chronic disease with links to various health issues, such as cardiovascular diseases, hormonal disorders, and cancers [114]. Hence, dietary choices play a pivotal role, positioning apples a crucial element in weight loss diets. These fruits are low in saturated fats and cholesterol, making them ideal for those battling obesity. Their low caloric content and a low glycemic index (GI) equal to 44 cause a slow and slight increase in blood sugar levels after consumption, which makes apples a recommended choice for managing weight. They protect against obesity and type 2 diabetes, as apple consumers tend to have lower body mass index (BMI) and a lower prevalence of obesity compared to non-consumers [44,115,116]. Apple polyphenols regulate leptin levels and influence visceral fat size, contributing to weight management [117]. Research on rats fed with 10% apple pomace showed protection against triglyceride deposition and lipid increase [118]. Kunkel et al. have shown that ursolic acid, found in apples, intensifies protein kinase B activity, which aids in glucose tolerance resistance, guarding against type 2 diabetes, obesity, and fatty liver disease. It enables glycolysis initiation and controls insulin-like growth factor 1 (IGF-1) signaling. Additionally, Akt kinase, present in skeletal muscles, promotes their growth and enhances their grip strength. Moreover, it increases the amount of brown adipose tissue, which helps maintain body temperature, as confirmed by the increased expression of the brown fat marker, uncoupling protein 1 [86].

The next component in apples gaining attention for its health benefits is dietary fiber, which is involved in proper stool formation and helps to avoid diarrhea or constipation [33]. In particular, insoluble fiber binds to and removes toxins from the body, including heavy metals, such as cadmium and lead, as well as insoluble salts [119,120]. Apple pectin fosters prebiotic activity and colonic microflora diversity, like *Bifidobacteria* or *Faecalibacterium prausnitzii* [121]. Compounds present in apples help to maintain a healthy body weight, promote weight loss, and prevent subsequent complications from excess body fat. Apples might also contain some amount of starch [122].

While apples contain valuable nutrients, like phenolic compounds, vitamins, and dietary fiber, their significant sugar content, especially fructose, warrants attention [123]. Fructose, a highly lipogenic sugar, holds a lower GI than glucose (GI = 20 vs. GI = 100) [123,124]. Fructose can be converted to glycerol 3-phosphate, which leads to increased synthesis of triglycerides in the liver [123]. This explains why long-term excessive consumption of fructose can contribute to non-alcoholic fatty liver disease [125]. Fructose overconsumption leads to leptin and insulin resistance, high blood pressure, and subsequent cardiovascular diseases [126,127]. Fructose is metabolized in the liver almost completely, and its excess is converted into fat. Special attention should be paid to consuming fruit juices, which are typically lacking in fiber. Fiber helps mitigate fructose absorption. That is why fiber should be consumed at the same time as fruit juices. Therefore, drinking large amounts of “healthy” juices may be misleading and contribute to increased body fat and mass. Fructose itself can also stimulate appetite. Thus, it is important not only to consume the right types of sugars, but also the right amount of them, adequate for the needs of the human organism. Awareness of the sugar content in fruits influence the determination of their acceptable intake. Given the high sugar content in apples, it is recommended not to exceed more than two apples per day. Keeping this in mind ensures that while reaping the benefits of apples, one does not inadvertently consume excess sugars that might lead to health complications [14,123,125].

## 5. Thermal Processing and Processed Apple Products

**Figure 7:**
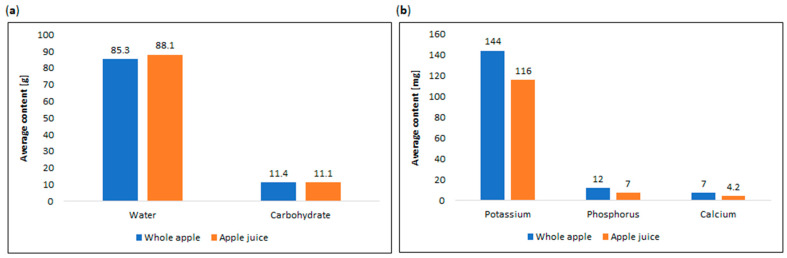
Comparison of average content of (**a**) water and carbohydrate in whole apple and apple juice, and (**b**) essential nutrients in whole apple and apple juice [56].

The versatile nature of apples allows for an array of consumed apple products, facilitated by fruit processing. Baking, cooking, drying, and juicing are common methods that transform apples into a convenient and nutritious snack. Apples may be utilized in the production of various items, including juice, wine, cider vinegar, spirits, syrup, sauce, puree, jam, and dried apple products. Additionally, cooked and chilled apple products remain popular and serve as a base for jams or desserts [122,128,129]. The disparity in essential nutrient content between whole apples and apple juice is evident in Figure 7 [56].

Most fruits are acidic and have a low pH, i.e., <4.5 (apples have a pH of 3.5), and for this reason, they need only mild pasteurization consisting of mild heat treatment, i.e., 50 °C (for apples, even 95 °C for about 20 min), which enhances microorganisms’ devastation, simultaneously preserving the taste of the products and avoiding deterioration of their nutritional value [122]. The quality of fruit and heat-processed fruit concern some aspects, like taste, aroma, and color, but the most important are nutritional values. Among the limiting factors related to the pasteurization process are physicochemical properties, like inactivation of heat resistant and endogenous enzymes. An example of such an enzyme is pectin methylesterase, an enzyme hydrolyzing the glycosidic bods of pectin [122,130]. These enzymes, highly thermos-resistant (activated at low temperatures 55–75 °C), determine the cloud stability of fruit juices through the presence of dissolved pectin colloids (~0.1 µm) and small particles (0.5–10.0 µm). Other food processing methods include cooking, drying, and frying [122]. These processing methods might have a certain impact on some physiochemical properties and shelf life, which can be elongated [131]. Heat treatment leads to slight modifications in the final processed product, notably softening of the fruit. Texture is closely related to sense of touch, and it may be checked by tested methods, including computer software, microscopic observations, and micromechanics. During heat treatment, texture changes are linked to pectin degradation, and that is why they depend on the treatment time, temperature degree, and activity of pectin degrading enzymes. During this process, cell membranes are destroyed, and cells lose their turgor pressure. Color degradation is also a result of thermal degradation of pigments. Moreover, juices from concentrates have higher amount of sulfur-containing substances due to thermal degradation of amino acids [122]. Processed products form slightly differently from the classic ones. Processed products are fortified with additional quantity of sugars but, simultaneously, they are deprived of the same amount of dietary fiber as raw fruit [44]. As a general rule, temperature escalates the soluble fibers that can lead to cell wall swelling [122]. The abundance of phytochemicals, particularly natural antioxidants, should be considered during the processing of apples, as high temperatures might be detrimental to bioactive compounds. The drying process, for instance, leads to the degradation of polyphenols, resulting in their reduced content in apples [131]. Freshly squeezed apple juice generally contains a higher concentration of polyphenols than juice prepared from concentrates: 154–970 mg/L and 110–459 mg/L, respectively. In comparison, a whole apple may contain 5230–27,240 mg/kg of total polyphenolic compounds [62]. Notably, Yuste et al. discovered that about 65% of phenols remain in fruits after the purée pasteurization process [132].

An important aspect is the decrease in vitamin C levels during the pasteurization process, which interlinks to less vitamin C in processed products [122]. Under normal conditions, one apple (about 100 g) has approximately 5.7 mg of pure vitamin C [50]. On the other hand, vitamin C is very often used in the processed products to inhibit enzymatic browning of products. Ascorbic acid is stable in anaerobic conditions and susceptible to degradation by oxidation. Fruits may contain both of these forms, but their content depend on particular cultivar, while dehydroascorbic acid (the oxidized form of vitamin C) is susceptible to heat degradation [122]. One apple product deserves special attention. Studies show the health-promoting role of apple cider vinegar, which contains 3–5% acetic acid. It also contains many polyphenols, vitamins, mineral salts, and amino acids. Produced via apple juice fermentation, this vinegar has shown beneficial effects on weight loss, diabetes, and atherosclerosis [117]. Apple pomace consists of various parts like flesh, peel, seeds, core, calyx, and some stem tissues. Notably, the flesh and other parts of fruit are rich in polyphenols, in particular seeds. Apple peel is another part of apple high in polyphenols and dietary fiber. Studies have shown that apple seed oil comprises even 90% of unsaturated fatty acids, considered as a reason of high antioxidant properties. This oil is an excellent source of oleic and linoleic acids [133]. Therefore, the processing of primary products lead to a reduction in the apple antioxidant capacity [122].

## 6. Conclusions

Given the diverse array of chemical compounds with health-enhancing properties, apples are a vital ingredient to a well-balanced diet, while their derived products can be a tasty snack. Their positive impact on the human organism, especially the circulatory system, is noteworthy. Apples (and their derivatives, to a lesser degree) represent a rich source of invaluable constituents that actively participate in cellular-level metabolic processes. This engagement helps maintain cellular homeostasis, ultimately aiding in the prevention or mitigation of numerous health issues, including notable diseases of civilization, such as cardiovascular diseases, cancers, diabetes, and obesity.

The consistent inclusion of apples in our diets deserves significant attention for their multifaceted benefits in promoting overall human health. Additionally, apples provide a safe, inexpensive, and readily available means of obtaining essential nutrients, such as vitamin C, quercetin, an array of antioxidants, and beneficial pectin. The various apple phytochemicals showcase substantial potential in both preventing and therapeutically addressing numerous health concerns.

## Figures and Tables

**Table 1:** Scientific classification of *Malus domestica* Borkhausen [27,28,29,30].

Taxonomic Unit	
Kingdom	*Plantae*
Family	*Rosaceae* Jussieu
Subfamily	*Amygdaloideae*
Tribe	*Maleae*
Subtribe	*Malinae*
Genus	*Malus* Miller
Section	*Malus*
Species	*Malus domestica* Borkhausen

## Abbreviations

AD	Alzheimer’s disease
AIDS	Acquired immunodeficiency syndrome
Aβ	Amyloid-β
Aβ42	Amyloid-β 42
Akt	Protein kinase B
ARE	Antioxidant response element
BMI	Body mass index
Borkh.	Borkhausen
CAT	Catalase
Ca^2+^	Calcium ions
CHUA	3β-trans-cinnamoyloxy-2α-hydroxy-urs-12-en-28-oic acid
CRC	Colorectal cancer
CuZnSOD	Copper zinc superoxide dismutase
CYP1A1	Cytochrome P450 family 1 subfamily A member 1
DNA	Deoxyribonucleic acid
EGFR	Epidermal growth factor receptor
ERK1/2	Extracellular signal-regulated kinases 1 and 2
FW	Fresh weight
GAPDH	Glyceraldehyde 3 phosphate dehydrogenase
GI	Glycemic index
GPx	Glutathione peroxidase
HCC	Hepatocellular carcinoma
HCl	Hydrochloric acid
HIV	Human immunodeficiency virus
H_2_O_2_	Hydrogen peroxide
IC_50_	Half-maximal inhibitory concentration
IGF-1	Insulin-like growth factor 1
LDL	Low-density lipoprotein
MAPK	Mitogen-activated protein kinases
MDA	Malondialdehyde
miRNA	Micro ribonucleic acid
MRSA	*Methicillin-resistant Staphylococcus aureus*
MT	Metric tons
NAD(P)H	Nicotinamide adenine dinucleotide (phosphate) hydrogen
NCDs	Non-communicable diseases
*NFE2L2*	*Nuclear factor erythroid-derived 2-like 2*
NF-κB	Nuclear factor kappa B
Nrf2	Nuclear factor erythroid 2-related factor 2
NMDA	*N*-methyl-D-aspartate
NO	Nitric oxide
OG	Oligogalactan
ORAC	Oxygen radical antioxidant capacity
OSCC	Oral squamous cell carcinoma
O_2_^-^	Superoxide anion
^·^OH	Hydroxyl radical
PA	Phloretamide
PCs	Procyanidins
*PGC-1*α	*Proliferator-activated receptor gamma coactivator 1-alpha*
Phldz	Phloridzin
Phlor	Phloretin
PI3K	Phosphoinositide 3 kinase
PPO	Polyphenol oxidase
ROS	Reactive oxygen species
SOD	Superoxide dismutase
SOD2	Superoxide dismutase 2
TE	Trolox equivalents
TLR-4	Toll-like receptor 4
UA	Ursolic acid
USA	United States of America
UV	Ultraviolet
5-FU	5-fluorouracil
